# Examining Sleep-Disordered Breathing Events Using Latent Profile Analysis

**DOI:** 10.1155/bn/8848485

**Published:** 2025-04-03

**Authors:** Marina Weinberger, Anwar E. Ahmed, Ahmed Almuttari, Abdullah Al-Harbi, Hani A. Alsaigh, J. Kent Werner, Hamdan Al-Jahdali

**Affiliations:** ^1^School of Medicine, Uniformed Services University of the Health Sciences, Bethesda, Maryland, USA; ^2^Department of Preventive Medicine and Biostatistics, Uniformed Services University of the Health Sciences, Bethesda, Maryland, USA; ^3^Division of Pulmonary, Sleep Disorders Center, King Abdulaziz Medical City/King Saud University for Health Sciences/King Abdullah International Medical Research Center, Riyadh, Saudi Arabia; ^4^College of Public Health and Health Informatics, King Saud bin Abdulaziz University for Health Sciences, Riyadh, Saudi Arabia; ^5^Department of Neurology, Uniformed Services University of the Health Sciences, Bethesda, Maryland, USA

**Keywords:** AHI_REM_/AHI_NREM_ ratio, obstructive sleep apnea, polysomnography, sleep

## Abstract

The clinical utility of the ratio of the apnea–hypopnea index (AHI) occurring during rapid eye movement (REM) and non-REM (NREM) sleep (AHI_REM_/AHI_NREM_ ratio) has been debated. We investigated the heterogeneity of REM and NREM sleep behaviors to identify unobserved distinct subtypes of sleep-disordered breathing (SDB) and examine their demographic and clinical features. The present study used a sample of 3626 adult patients who underwent diagnostic polysomnography evaluations at the Sleep Disorders Center of King Abdulaziz Medical City in Riyadh, Saudi Arabia. Latent profile analysis was performed to categorize subjects into distinct profiles of SDB based on AHI_REM_, AHI_NREM_, and AHI_REM_/AHI_NREM_ ratio. A multinomial logistic model estimated the odds ratio of SDB profiles. Four distinct subtypes of SDB were identified: Class I (low AHI_REM_; 75.9%) included patients with normal SDB events during REM sleep, serving as the reference group; Class II (REM-OSA, 1.2%) included patients with high AHI during REM sleep but lowest AHI during NREM sleep, resulting in the largest AHI_REM_/AHI_NREM_ ratio; Class III (AHI_NREM_ < 30 events per hour, 17.4%); and Class IV (AHI_NREM_ ≥ 30 events per hour, 5.5%). Compared to Class I, factors related to Class IV included older age, high BMI, large neck circumference, hypertension, reduced total sleep time, reduced REM sleep, poor sleep efficiency, high desaturation index, low SpO2, high arousal index, and high Epworth Sleepiness Scale. As hypothesized, the study characterized several subtypes of SDB based on the AHI_REM_, AHI_NREM_, and their ratio (AHI_REM_/AHI_NREM_) in a large cohort and identified their demographic and clinical features. These subtypes might be clinically useful for defining SDB among adult patients referred to sleep clinics who may have varying responses to treatment depending on their subtype of the disease.

## 1. Introduction

Obstructive sleep apnea (OSA) is a widespread disease of sleep-disordered breathing (SDB) [1]. In North America, it is estimated that 15%–30% of men and 10%–15% of women have OSA [2], with a total worldwide prevalence estimated at 936 million people [3]. OSA is caused by a collapse of the soft tissues of the upper airway during sleep, which leads to partial or complete reductions in airflow (known as hypopneas and apneas, respectively) that must last at least 10 s but typically are between 10 and 30 s [4]. The severity of OSA is generally measured using the apnea–hypopnea index (AHI), a representation of the average number of sleep disturbance events in 1 h of sleep. The American Academy of Sleep Medicine defines a person without OSA as having an AHI of < 5, mild OSA of 5–15, moderate OSA of 15–30, and severe OSA > 30 events per hour [4].

There has been extensive debate in the past four decades as to whether there is clinical significance of OSA occurring during the rapid eye movement (REM) phase of sleep versus the nonrapid eye movement (NREM) phase of sleep. Early research showed that respiratory events were longer during REM sleep than NREM sleep in OSA patients [5], leading to the notion that OSA had a stronger relationship to REM. However, subsequent studies have shown that apnea events that occur during NREM sleep are associated with excessive daytime sleepiness and impaired quality of life in OSA patients, while apnea events during REM sleep did not show these associations [6, 7].

As the clinical significance of REM-related OSA has been scrutinized, attempts have been made to standardize its definition. The most widely used definition is a AHI_REM_/AHI_NREM_ ratio of ≥ 2, which indicates that the majority of a patient's SDB occurs during REM sleep [1]. However, several limitations to this method have been exposed [1], primarily in the binary classification between REM-OSA and NREM-OSA; this ratio-based approach is a binary classification that overlooks the diversity of subtypes that can occur within SDB, such as central, postarousal, arousal-promoting, or desaturation-promoting events. Nevertheless, even the phenotypic classification of REM-OSA versus NREM-OSA may be clinically useful as treatment, outcomes may depend on the patient's phenotypic subtype of OSA. For example, one study has shown that mandibular advancement splints (MASs) have a significantly lower treatment response rate in patients with REM-predominant OSA over NREM or nonstage dependent OSA, with complete resolution of symptoms occurring in only 12% of REM-OSA patients as opposed to other subtypes (42% in NREM and 32% in nonstage dependent). This implies a need for reliable diagnostics to categorize patients with OSA into phenotypic subtypes to better predict treatment outcomes. Notably, the reproducibility of this finding has been mixed as other research has demonstrated no significant difference in MAS treatment outcomes between REM and nonstage-specific OSA. This could be due to patient heterogeneity, the smaller sample size, and the exclusion of NREM-predominant OSA in the analysis. Therefore, while the REM/NREM ratio is a useful metric, a multidimensional classification approach would provide a more comprehensive and clinically relevant method for classifying OSA subtypes.

Latent profile analysis (LPA) is one method we propose to integrate multiple variables to identify distinct subgroups, allowing for a more nuanced classification of SDB phenotypes than the current standard. In the present study, we used LPA to identify and characterize distinct subtypes of SDB within a large cohort of heterogenous patients based on AHI-REM, AHI-NREM, and the AHI-REM/AHI-NREM ratio. We hypothesized that a data-driven classification of patients who were referred for evaluation of OSA would identify subgroups of increased risk for REM or NREM-AHI predominant OSA which vary by their demographic and clinical features. The aim of this study was to identify unobserved distinct subtypes of SDB and examine their demographic and clinical features in patients who were referred for evaluation of OSA and underwent overnight PSG. This study, the first to apply LPA to the AHI_REM_/AHI_NREM_ ratio, offers a patient-centered alternative to traditional statistical methods by identifying latent subgroups that may inform targeted interventions.

## 2. Materials and Methods

### 2.1. Study Design

A retrospective chart review was conducted from December 2003 to May 2023 at the Sleep Disorders Center (SDC) of King Abdulaziz Medical City in Riyadh (KAMC-R), a 2000-bed hospital in Saudi Arabia. The SDC is considered one of the largest tertiary hospitals in the Middle East and is staffed with several physicians and certified sleep technologists trained to diagnose sleep apnea and other sleep disorders. The center has maintained a patient registry since the SDC's opening in 2003. Ethical approval for this study was granted by the Ministry of National Guard-Health Affairs, Institution of Research Board (IRB) registered under No. RC15/058/R.

### 2.2. Participants

The analysis included 6883 patients who underwent polysomnography during the study period. We excluded patients aged younger than 18 years (*n* = 524), all therapeutic sleep studies, narcolepsy, less than 3 h of total sleep time, and less than 30 min of REM sleep time (*n* = 2733). Requiring at least 30 min of REM sleep ensured that our findings were reliable, generalizable, and clinically relevant. The 30 min enabled the capture of stable REM sleep, which is characterized by REMs, increased brain activity, and dreams [8]. The final analytic sample was 3626.

### 2.3. Measurements

We retrieved data on demographics, comorbidities, sleepiness scale as per the Epworth Sleepiness Scale (ESS) [9, 10], sleep study results, and final diagnosis. All patients in this study underwent standard in-lab Type I PSG. PSGs were recorded using Alice 5 and Alice 6 diagnostic equipment (Respironics Inc., Murrysville, Pennsylvania, United States). Expert certified sleep technologists performed manual scoring of the electronic data by following established criteria [11]. The sleep time was measured using a split study in which one half was comprised of diagnostic polysomnographic studies (measured as thermal sensor excursion detected via an oronasal thermal sensor) and the other half of PAP titration studies (measured as positive airway pressure device flow). Hypopnea was defined as a ≥ 30% reduction in airflow for at least 10 s that caused either a ≥ 3% drop in oxygen saturation or arousal. Apnea was defined as a ≥ 90% reduction in peak thermal sensor excursion for at least 10 s. An apneic event was identified by obstructive apnea in the presence of continued respiratory effort. The study's outcome was the AHI, which was categorized as follows: normal (AHI < 5) per hour, mild (5 ≥ AHI < 15) per hour, moderate (15 ≥ AHI < 30) per hour, and severe (AHI ≥ 30) per hour [11]. Each patient underwent polysomnography to assess sleep parameters including total sleep time, total time of NREM and REM sleep, sleep efficiency, sleep stage percentages (N1, N2, and N3), stage REM (%), arousal index, desaturation index, lowest SpO2 (%), wake after sleep onset, and latency to persistent sleep. Further details of the study design and data collection can be found in our recent publication [12].

### 2.4. Statistical Analysis

The LPA was conducted in Mplus Version 8.8 (Muthén & Muthén, 1998–2022) to define distinct SDB profiles. The three variables AHI_REM_, AHI_NREM_, and the ratio AHI_REM_/AHI_NREM_ were included to estimate the conditional probability of SDB and to assign each patient to a subtype of SDB. LCA solutions were compared according to the elbow plot of the Bayesian Information Criterion (BIC) and Akaike's information criterion (AIC). An optimal number of profiles was determined based on the likelihood ratio test. Data were presented as frequency and percent and mean and standard deviation (Table 1). Associations between SDB profiles and demographic and clinical factors were assessed using chi-square for percentages or ANOVA/Kruskal–Wallis for means (Table 2). A multinomial logistic regression was used to model SDB profiles (Table 3). Class I (low AHI_REM_) was used as the reference category. To avoid including correlated covariates in one model, we ran a separate multinomial logistic model for each clinical and polysomnographic parameter (e.g., total sleep time and total sleep time NREM) while controlling for demographic characteristics. Univariate, subgroup, and adjusted analyses were performed using SAS software Version 9.4 (SAS Institute Inc., Cary, North Carolina).

## 3. Results

### 3.1. Demographic and Health Characteristics

Of the 3626 adult patients who underwent diagnostic polysomnography evaluations, 1756 (48.4%) were female, and the mean age was 50.1 ± 14.6 (range 18–93) years (Table 1). The mean neck circumference for women was 39.0 ± 3.6 cm (range 28–53 cm) and for men was 42.1 ± 3.6 cm (range 27–54 cm). Of the study cohort, 82% of the patients were obese (22.2% obesity Class I, 19.3% Class II, and 40.5% Class III). In our sample, 1094 patients (30.2%) were categorized as normal OSA (AHI < 5), 747 (20.6%) were mild OSA (5 ≥ AHI < 15), 615 (17.0%) were moderate OSA (15 ≥ AHI < 30), and 1170 (32.2%) were severe OSA (AHI ≥ 30).

### 3.2. SDB Profiles

**Figure 1:**
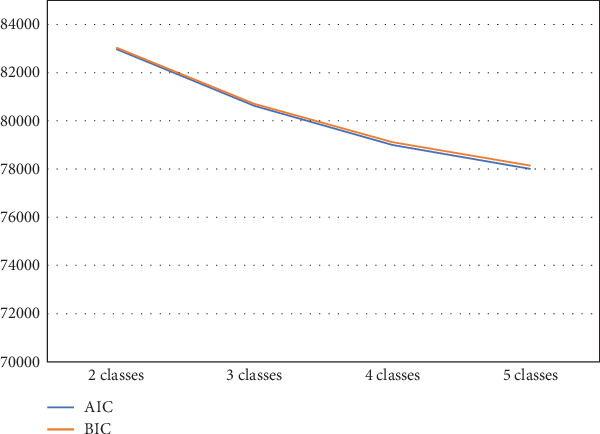
Goodness of fit across different number of classes.

**Figure 2:**
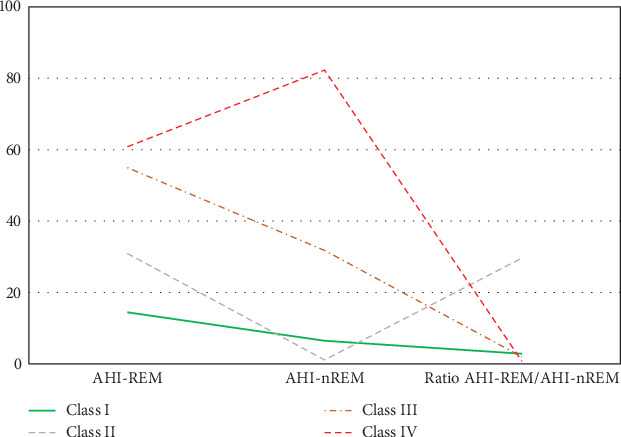
Estimated means of AHIREM, AHINREM, and the ratio AHIREM/AHINREM in each of the four classes. Most patients (75.9%) who were referred to the sleep clinic were categorized as Class I “low AHIREM,” with lowest AHIREM. Patients with low AHINREM but a high ratio were categorized as Class II “REM-OSA” (1.2%). Class III patients had intermediate AHINREM “AHINREM < 30 events per hour” (17.4%). Class IV patients had high AHINREM “AHINREM ≥ 30 events per hour” (5.5%).

The elbow plot reveals that for both the BIC and AIC indices, a sharp decline stops at the four-class solution (Figure 1). We also used the log-likelihood ratio test to determine the optimal number of classes based on several parameters: (1) The *p* value was smaller than a significance value of 0.05 for 3 (H_0_) versus 4 classes (H_1_) (*p* = 0.0197), and (2) the *p* value was higher than a significance value of 0.05 for 4 (H_0_) versus 5 classes (H_1_) (*p* = 0.1114). Thus, a four-class solution was determined to be most appropriate to fit the data. As hypothesized, the LPA identified a four-class solution based on the probabilities of SDB (Figure 2). Class I (*n* = 2753, 75.9%) includes patients with minimal probabilities of SDB. Class I can be characterized as patients with the lowest value for AHI_REM_ (low AHI_REM_). Class II (*n* = 42, 1.2%) includes patients with the lowest AHI_NREM_ and highest ratio (REM-OSA). Class III (*n* = 633, 17.4%) includes patients with intermediate values for AHI_NREM_ (AHI_NREM_ < 30 events per hour). Class IV (*n* = 198, 5.5%) characterizes patients with the highest AHI_NREM_ (AHI_NREM_ ≥ 30 events per hour).

### 3.3. Subgroup Analysis

Table 1 describes sample characteristics and SDB profiles. Table 2 illustrates prevalence estimates of comorbidities by SDB classes. Compared to Class II (REM-OSA), the prevalence estimates of hypertension, asthma, and diabetes were significantly higher in Class III (AHI_NREM_ < 30 events per hour) and Class IV (AHI_NREM_ ≥ 30 events per hour). Table 2 also illustrates the mean estimates of demographic characteristics and polysomnographic measurements by SDB classes. The mean estimates of age, BMI, and neck circumference increased systematically with Class II (REM-OSA), Class I (low AHI_REM_), Class III (AHI_NREM_ < 30 events per hour), and Class IV (AHI_NREM_ ≥ 30 events per hour). The mean estimates of the ESS and all polysomnography indices (except Stage N1%) significantly varied by the SDB classes.

### 3.4. Adjusted Analysis

The independent associations between demographic and clinical characteristics and the odds of SDB profiles were evaluated with a multinomial logistic model (Table 3). Compared to Class I (low AHI_REM_), Class IV (AHI_NREM_ ≥ 30 events per hour) was predicted by older age, higher BMI, larger neck circumference, hypertension, reduced total sleep time, reduced REM sleep, poor sleep efficiency, higher Stage N2%, higher REM Stage %, higher arousal index, higher desaturation index, lower SpO2 (%), higher wake after sleep onset, higher latency to persistent sleep, and higher ESS. Compared to Class I (low AHI_REM_), Class III (AHI_NREM_<30 events per hour) was predicted by older age, larger neck circumference, lower total sleep time, lower total sleep time REM, higher Stage N3%, higher REM stage %, higher arousal index, higher desaturation index, and lower SpO2 (%). Compared to Class I (low AHI_REM_), Class II (REM-OSA) was negatively associated with asthma, total sleep time, and desaturation index.

## 4. Discussion

The question of whether OSA is more severe during REM or NREM sleep has been extensively debated in the literature. Physiologically, it is expected that REM sleep would increase the severity of OSA due to REM atonia, which produces inhibition of upper airway muscle activation [13] and decreased genioglossus reflex in response to negative airway pressure during REM sleep [14]. However, some studies have shown the opposite: The expected symptoms of OSA (excessive daytime sleepiness and impaired quality of life) are actually worse in patients with elevated AHI_NREM_ [6, 7]. Thus, the AHI_REM_/AHI_NREM_ ratio is one tool that has been explored as an alternative method to interrogate this question [15].

**Figure 3:**
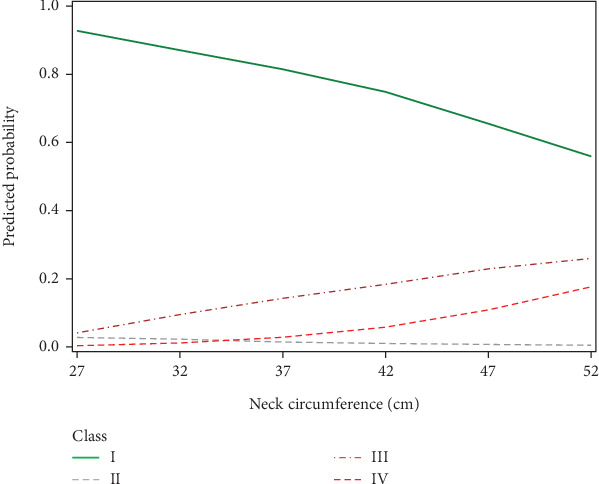
Differences in neck circumference values by SDB profiles. The predicted probability of being in Class III “AHINREM < 30 events per hour” and in Class IV “AHINREM ≥ 30 events per hour” increased with neck circumference values.

**Figure 4:**
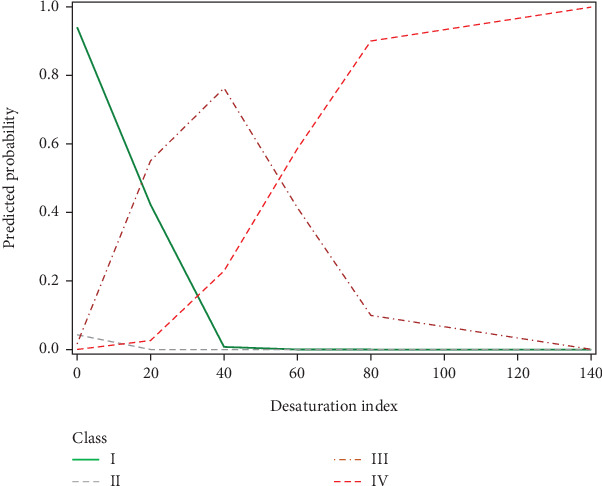
Differences in desaturation index values by SDB profiles. The predicted probability of being in Class IV “AHINREM ≥ 30 events per hour” increased with desaturation index values. The predicted probability of being in Class III “AHINREM < 30 events per hour” increased with desaturation index values less than 40 and decreased with desaturation index values greater than 40. The predicted probability of being in Class I “low AHIREM” decreased with desaturation index values.

**Figure 5:**
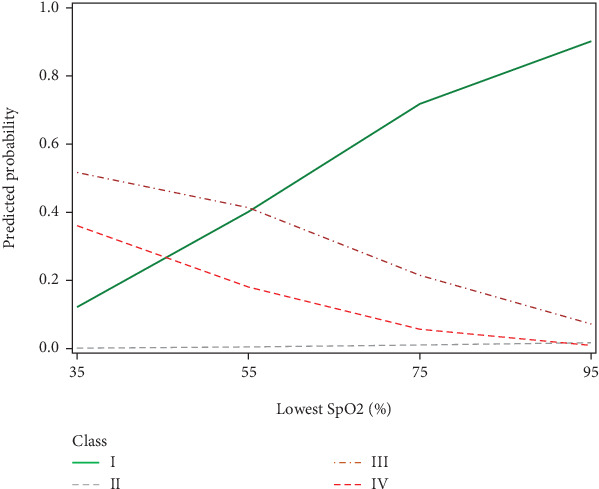
Differences in lowest SpO2 (%) by SDB profiles. The predicted probability of being in Class III “AHINREM < 30 events per hour” and in Class IV “AHINREM ≥ 30 events per hour” decreased with SpO2 (%) values. The probability of being in Class I “low AHIREM” increased with SpO2 (%) values.

In the present study, we divided patients undergoing evaluation for OSA into distinct profiles of SDB using LPA and compared their means of AHI_REM_, AHI_NREM_, and AHI_REM_/AHI_NREM_ ratio (Figure 2). Four profiles were chosen based on the abovedesired characteristics. In Class II (referred to as REM-OSA), we noted high AHI_REM_ with the lowest AHI_NREM_ and the highest ratio of AHI_REM_/AHI_NREM_. This class was unique because it was not predicted by neck circumference (Figure 3) and oxygen desaturation index (ODI) (Figures 4 and 5), suggesting obesity may have a stronger association with NREM-OSA than REM-OSA.

The relationship between age and REM versus NREM SDB is a central consideration of this paper. In general, the literature supports a continuous increase in the risk of OSA with age [16]. However, REM-associated OSA has been shown to be more common among younger people [14], while NREM-associated OSA may be more common in older populations [11]. It is hypothesized that this may be due to NREM sleep being associated with greater respiratory effort than REM sleep [17], as airway tone is lost with age, and SDB becomes more apparent during the NREM stage of sleep. This hypothesis would support our finding that older age was associated with SDB in Classes III and IV, which have intermediate and high values of apnea during NREM sleep, respectively.

OSA has been widely shown to be related to obesity, as excess body tissue contributes to compression of the airway during sleep [18]. This relationship may be bidirectional: Obesity has been shown to increase the risk for SDB (e.g., a 10% weight gain is associated with a 6-fold increase in odds of developing OSA [19]), while OSA may also increase the odds of developing obesity, likely through a multifactorial association involving predisposition to weight gain due to daytime somnolence leading to decreased exercise and endocrine dysfunction [18]. Our SDB profiles supported this relationship as well, as demonstrated by the increase in the probability of SDB Class IV with increasing BMI. The probability of being in Classes III and IV was also positively associated with neck diameter (Figure 3), further reinforcing this relationship.

Another parameter evaluated in the present study was the ODI, a measurement that has been proposed as an alternative to PSG for use in grading OSA severity [19]. Interestingly, we found unique patterns of ODI values between the four SDB profiles (Figure 4). The probability of being in Class I (low AHI_REM_) decreased and the probability of being in Class IV increased with ODI, as anticipated [20]. However, the probability of being in Class III followed a similar trend to Class IV before an ODI of 40 and then followed a similar trend to Class I at index values higher than 40, suggesting a mean ODI of 40 among moderate-severity OSA patients. This finding is in agreement with other studies, which demonstrated ODI cutoff values of 25 had 89.7% sensitivity, and 20 had 96.5% sensitivity for severe OSA [21]. Our findings suggest that a cutoff value in this range may be clinically useful for the development of a screening test for identifying possible cases of moderate to severe OSA. This would be especially relevant for developing the ODI into a screening test, as it is more cost-effective and requires less equipment to carry out than the traditional PSG test.

We further investigated SpO_2_ measurements as another parameter for OSA risk. We found that the probability of being in Classes III and IV increased with decreasing SpO_2_ nadir and the low probability of SDB (Class I) increased with increasing SpO_2_ nadir (Figure 5). This pattern is consistent with previous studies which have shown that SpO_2_ nadir is a predictor of OSA severity [22]. Interestingly, the mean lowest SpO_2_ for mild, moderate, and severe OSA in the comparison study were 95.7%, 95.7%, and 93.6% [19], which were all significantly higher than the lowest SpO_2_ values for the present study (83.6%, 87.5%, 74.2%, and 65.8% for Classes I–IV, respectively). This discrepancy indicates that further research is needed to interrogate SpO_2_ as a reliable predictor of OSA risk.

Arousal index was also found to be associated with Classes III and IV, whose probability increased with this parameter. Arousal index was highest in Class IV (mean = 61.2), followed by Class III (mean = 29.4). Classes I and II were similar, with arousal indices of (mean = 17.0 and mean = 15.9, respectively). As the arousal index is a polysomnographic indicator of disrupted sleep, these results support the association of poorer sleep quality with REM-OSA. These findings are also in line with literature that supports the arousal index being lower in both less severe OSA and REM-predominant OSA [23, 24].

The ESS is an additional measure evaluated in the present study, which has been widely used for the past two decades to measure daytime sleepiness to aid in the diagnosis of sleep disorders [10]. We found that higher values scored on the ESS were associated with a higher probability of Class IV, further supporting that high NREM-associated OSA with a low AHI_REM_/AHI_NREM_ ratio is indicative of more severe disease. Contrary to our findings, Gabryelska and Białasiewicz [25] have found similar ESS scores between REM-OSA and NREM-OSA groups despite presenting with different AHI levels. However, they did not find any group differences in SpO_2_ nadir or arousal index, suggesting that these parameters may explain the effect in ESS that was observed in the present study.

Lastly, we found that total sleep time was negatively associated with the probability of Classes III and IV. This finding provides evidence that the higher the proportion of OSA occurring during NREM sleep, the poorer the sleep duration and, thus, the greater the impact of OSA on sleep health. This finding also contradicts the findings of Gabryelska and Białasiewicz [25], who did not find differences in total sleep time between REM-OSA and NREM-OSA. However, the previously stated differences in other parameters may explain the lack of an effect seen on sleep duration.

The present study has several strengths, such as its relatively large sample size of 3626 participants which lends validity to our results. The use of a multinomial logistic model with LPA enabled risk evaluations to be made for four distinct classes of SDB, each with independent associations with various PSG parameters as discussed above.

We acknowledge that this study presents some limitations as well. The study cohort was comprised of individuals who were referred to a sleep clinic and had several comorbidities (82% obese, 46.4% hypertension, and 39.7% Type 2 diabetes mellitus) that are not representative of the general population, thus conferring a possible selection bias. Thus, our findings are most applicable to patients who are already being evaluated for possible SDB. Additionally, the cross-sectional nature of this study also confers certain limitations, most notably in the inability to infer causality between NREM-OSA and the various polysomnographic parameters discussed above. The sleep time was measured during diagnostic and titration studies which might lower the sleep time due to adjustment of the treatment device. This may be due to microawakenings caused by gradual increases in the air pressure that occur during the titration process of the study, as well as general discomfort from wearing the CPAP mask possibly contributing to a shortened sleep time [11].

## 5. Conclusion

This study demonstrated a strong relationship between NREM-predominant OSA (AHI_NREM_ < 30 events per hour and AHI_NREM_ ≥ 30 events per hour) with various PSG parameters, including older age, increased BMI and neck circumference, decreased SpO_2_ nadir, increased arousal index, higher ESS values, and lower total sleep time. Interestingly, ODI for the intermediate NREM group (AHI_NREM_ < 30 events per hour) was found to follow low-NREM patterns below 40, and high-NREM (AHI_NREM_ ≥ 30 events per hour) patterns above 40. Further research in this area could focus on developing the desaturation index into a predictor of OSA severity.

## Figures and Tables

**Table 1:** Sample characteristics and sleep-disordered breathing (SDB) profiles.

		**n**	**%**

SDB profiles	Class I: low AHI_REM_	2753	75.90%
	Class II: REM-OSA	42	1.20%
	Class III: AHI_NREM_ < 30	633	17.50%
	Class IV: AHI_NREM_ ≥ 30	198	5.50%
Gender	Female	1756	48.40%
	Male	1869	51.60%
HTN	No	1944	53.60%
	Yes	1682	46.40%
CAD	No	3509	96.80%
	Yes	117	3.20%
CHF	No	3462	95.50%
	Yes	164	4.50%
BA	No	2788	76.90%
	Yes	838	23.10%
COPD	No	3416	94.20%
	Yes	209	5.80%
DM	No	2188	60.30%
	Yes	1438	39.70%

		**Mean**	**SD**

Age		50.1	14.6
BMI		38.4	11
Neck size		40.6	3.9
Total sleep time		287.1	80.7
Total sleep time non-REM		232.8	64.8
Total sleep time REM		55.7	70.7
Sleep efficiency		74	17.6
Stage N1%		17.7	24.9
Stage N2%		27.9	35.1
Stage N3%		58.6	71.8
REM stage %		108.1	83.3
Arousal index		21.4	17.2
Desaturation index		11.7	17.9
Lowest SpO2 (%)		81.1	15.1
Wake after sleep onset		77.3	56.7
Latency to persistent sleep		27.5	40.8
Epworth Sleepiness Scale		9.9	5.6
AHI_REM_		24.4	26.6
AHI_NREM_		15.1	20.6
Ratio AHI_REM_/AHI_NREM_		2.9	4

*Note:* N1%, N2%, N3%, and REM stage % are the duration of each stage to the total sleep time × 100%. We interpret each sleep stage as the average duration of the stage and summarized by mean and standard deviation rather than percentages.

Abbreviations: BA, bronchial asthma; CAD, coronary artery disease; CHF, congestive heart failure; COPD, chronic obstructive pulmonary disease; DM, diabetes mellitus; HTN, hypertension.

**Table 2:** Prevalence of SDB profiles by demographic and clinical characteristics.

		**Class I**		**Class II**		**Class III**		**Class IV**		
		**Low AHI **_**REM**_		**REM-OSA**		**A** **H** **I**_**N** **R** **E** **M**_ < 30		**A** **H** **I**_**N** **R** **E** **M**_ ≥ 30		
		**n**	**%**	**n**	**%**	**n**	**%**	**n**	**%**	**p**

Male	No	1350	49.1	23	54.8	305	48.2	78	39.4	0.055
	Yes	1402	50.9	19	45.2	328	51.8	120	60.6	
HTN	No	1539	55.9	28	66.7	299	47.2	78	39.4	< 0.001
	Yes	1214	44.1	14	33.3	334	52.8	120	60.6	
CAD	No	2676	97.2	41	97.6	604	95.4	188	94.9	0.056
	Yes	77	2.8	1	2.4	29	4.6	10	5.1	
CHF	No	2641	95.9	40	95.2	593	93.7	188	94.9	0.103
	Yes	112	4.1	2	4.8	40	6.3	10	5.1	
BA	No	2099	76.2	40	95.2	503	79.5	146	73.7	0.007
	Yes	654	23.8	2	4.8	130	20.5	52	26.3	
COPD	No	2604	94.6	39	92.9	592	93.5	181	91.4	0.222
	Yes	148	5.4	3	7.1	41	6.5	17	8.6	
DM	No	1722	62.5	30	71.4	330	52.1	106	53.5	< 0.001
	Yes	1031	37.5	12	28.6	303	47.9	92	46.5	

		**Mean**	**SD**	**Mean**	**SD**	**Mean**	**SD**	**Mean**	**SD**	

Age		49.3	14.5	47.6	17.2	52.9	14.5	54	13.3	< 0.001
BMI		38	11.3	36.6	10	39.6	9.5	41.1	10.5	< 0.001
Neck size		40.3	3.8	39.6	3.5	41.3	3.8	42.6	3.8	< 0.001
Total sleep time		292.4	79.6	281.8	76.2	276.9	74.6	245.6	99.4	< 0.001
Total sleep time non-REM		234.5	63.8	236.7	57.5	230.2	63	215.6	82.1	0.007
Total sleep time REM		59.6	78.9	45.6	34.9	47.1	29.3	29.8	26	< 0.001
Sleep efficiency		75	17.3	73.8	13.9	72.7	16.7	63.7	21.9	< 0.001
Stage N1%		17.5	24.3	21.7	31.1	18.1	25.2	18	30.2	0.689
Stage N2%		26.6	33	31.9	36	29	35.6	42	54.2	< 0.001
Stage N3%		56.9	69.4	70.1	81.2	66.3	78	57.2	83.5	0.003
REM stage%		100.9	79.1	121.9	88.1	127.7	85.7	144.5	109.9	< 0.001
Arousal index		17	11.8	15.9	10.5	29.4	15.3	61.2	26.5	<0.001
Desaturation index		5.1	6.9	0.9	1.4	25.2	12.8	65.7	26.6	< 0.001
Lowest SpO2 (%)		83.6	10.8	87.5	7.4	74.2	24.2	65.8	14	< 0.001
Wake after sleep onset		74	55.3	74.1	46.5	80.9	53.5	114.4	73.3	< 0.001
Latency to persistent sleep		26.4	38.8	24.5	33.7	27.9	40	41.6	64	0.047
Epworth Sleepiness Scale		9.9	5.6	8.5	6.4	9.8	5.3	11.4	5.3	0.002

Abbreviations: BA, bronchial asthma; CAD, coronary artery disease; CHF, congestive heart failure; COPD, chronic obstructive pulmonary disease; DM, diabetes mellitus; HTN, hypertension.

**Table 3:** Associations between sample and clinical characteristics and SDB profiles.

	**SDB profiles (** **r** **e** **f** = **Class** **I)**								
	**Class II**			**Class III**			**Class IV**		
	**REM-OSA**			**A** **H** **I**_**N** **R** **E** **M**_ < 30			**A** **H** **I**_**N** **R** **E** **M**_ ≥ 30		
	**OR**	**LCL**	**UCL**	**OR**	**LCL**	**UCL**	**OR**	**LCL**	**UCL**
Female	1.578	0.655	3.802	1.023	0.815	1.285	0.754	0.527	1.081
Age	0.991	0.969	1.014	**1.017**	**1.011**	**1.024**	**1.026**	**1.015**	**1.037**
BMI	0.975	0.931	1.021	1.007	0.997	1.017	**1.014**	**1.001**	**1.026**
Neck size	0.998	0.891	1.118	**1.067**	**1.037**	**1.098**	**1.138**	**1.089**	**1.189**
HTN	0.595	0.275	1.287	1.066	0.868	1.31	**1.44**	**1.018**	**2.038**
CAD	1.012	0.134	7.671	1.316	0.834	2.077	1.139	0.548	2.368
CHF	1.507	0.341	6.65	1.165	0.782	1.735	0.863	0.432	1.724
BA	**0.161**	**0.038**	**0.679**	**0.729**	**0.581**	**0.914**	1.202	0.848	1.704
COPD	1.73	0.492	6.084	0.788	0.531	1.17	1.092	0.621	1.918
DM	0.607	0.275	1.338	1.18	0.962	1.448	0.987	0.703	1.386
Total sleep time	0.999	0.995	1.003	**0.998**	**0.997**	**0.999**	**0.994**	**0.993**	**0.996**
Total sleep time non-REM	1.001	0.996	1.006	1	0.999	1.002	0.998	0.996	1
Total sleep time REM	**0.988**	**0.977**	**0.999**	**0.988**	**0.985**	**0.991**	**0.965**	**0.958**	**0.971**
Sleep efficiency	0.997	0.979	1.016	0.996	0.99	1.001	**0.974**	**0.966**	**0.981**
Stage N1%	1.002	0.991	1.014	1.001	0.998	1.005	1.002	0.996	1.008
Stage N2%	1.002	0.993	1.011	1.002	1	1.005	**1.008**	**1.005**	**1.012**
Stage N3%	1.003	0.999	1.007	**1.002**	**1.001**	**1.003**	1.001	0.998	1.003
REM stage %	1.003	0.999	1.006	**1.004**	**1.003**	**1.005**	**1.007**	**1.005**	**1.008**
Arousal index	0.988	0.952	1.025	**1.079**	**1.07**	**1.087**	**1.14**	**1.127**	**1.153**
Desaturation index	**0.565**	**0.437**	**0.729**	**1.244**	**1.223**	**1.265**	**1.359**	**1.329**	**1.388**
Lowest SpO2 (%)	1.006	0.997	1.015	**0.942**	**0.935**	**0.95**	**0.918**	**0.908**	**0.927**
Wake after sleep onset	1.001	0.995	1.007	1.001	1	1.003	**1.009**	**1.007**	**1.012**
Latency to persistent sleep	0.995	0.985	1.006	1.001	0.999	1.003	**1.005**	**1.003**	**1.008**
Epworth Sleepiness Scale	0.973	0.913	1.036	0.988	0.971	1.005	**1.032**	**1.003**	**1.061**

*Note:* Boldface indicates statistical significance (*p* = 0.05).

Abbreviations: BA, bronchial asthma; CAD, coronary artery disease; CHF, congestive heart failure; COPD, chronic obstructive pulmonary disease; DM, diabetes mellitus; HTN, hypertension.

## Data Availability

The data that support the findings of this study are available from the corresponding author upon reasonable request and approval from King Abdullah International Medical Research Center, Riyadh, Saudi Arabia.

## Nomenclature

AHI: apnea–hypopnea index
SDB: sleep-disordered breathing
OSA: obstructive sleep apnea
ODI: oxygen desaturation index
PSG: polysomnography
